# Effect of an additional gonadotropin-releasing hormone 2 days after the initiation of a resynchronization program 25 days after artificial insemination on fertility of lactating dairy cows

**DOI:** 10.3168/jdsc.2023-0540

**Published:** 2024-04-20

**Authors:** Iago Matheus Rosa Leão, Anthony Carbajal, César Narciso, Carlos Eduardo Cardoso Consentini, Roberto Sartori, João Paulo Nascimento Martins

**Affiliations:** 1 Department of Medical Sciences, School of Veterinary Medicine, University of Wisconsin–Madison, Madison, WI 53706; 2 Sequoia Veterinary Services, Tulare, CA 93274; 3 Department of Animal Sciences, Escola Superior de Agricultura Luiz de Queiroz, Universidade de São Paulo, Piracicaba, SP, Brazil, 13418

## Abstract

We investigated the effect of an additional GnRH 2 d after initiation of resynchronization on d 25 post-AI on the proportion of cows without a corpus luteum (CL) at nonpregnancy diagnosis (NPD) and pregnancy per AI (P/AI) of cows with a CL at NPD that were reinseminated. Once per week, cows that were inseminated 25 d earlier were randomly assigned to receive either a GnRH treatment only (G25) or GnRH on this day and 2 d later (G25&27). Pregnancy diagnosis and CL presence were determined d 32 post-AI using ultrasonography. Nonpregnant cows with a CL were enrolled in different strategies for resynchronization of ovulation according to parity (primiparous or multiparous) and CL size. Primiparous and multiparous cows with a CL ≥20 mm in diameter continued Resynch-25 (PGF_2α_ on d 32; PGF_2α_ on d 33; GnRH on d 34; and AI on d 35). Multiparous cows with CL <20 mm restarted a modified Ovsynch at NPD (GnRH – 7 d – PGF_2α_ – 1 d – PGF_2α_ – 1 d – GnRH – 1 d – AI). Primiparous cows with CL diameter <20 mm started an Ovsynch+CIDR protocol on the day of NPD (GnRH + CIDR inserted – 7 d – PGF_2α_ + CIDR removed – 1 d – PGF_2α_ – 1 d – GnRH – 1 d – AI). Cows without a CL were not enrolled in any resynchronization program for a week and were excluded from fertility analyses. In multiparous cows only, the additional d 27 GnRH treatment was associated with a decreased P/AI 32 d and 102 d post-AI for the AI before treatment. The overall proportion of nonpregnant cows without CL did not differ between treatments. Yet, the GnRH treatment 27 d after AI tended to increase the proportion of overall nonpregnant cows with CL <20 mm at NPD. Treatment did not affect fertility parameters of primiparous cows with a CL ≥20 mm that continued Resynch-25 at NPD. Primiparous cows in G25&27 with CL <20 mm and submitted to Ovsynch+CIDR at NPD tended to have decreased P/AI 102 d after AI and calving/AI. When fertility parameters of primiparous cows with a CL independent of size at NPD were analyzed combined, no effect of treatment was observed. Treatment did not affect fertility of multiparous cows with CL <20 mm enrolled in Ovsynch at NPD. Multiparous cows in the G25&27 group that had a CL ≥20 mm and continued Resynch-25 at NPD tended to have a greater calving/AI due to a decreased total pregnancy loss. In summary, no treatment effect was observed on the overall fertility of primiparous cows with CL at NPD. In multiparous cows, G25&27 was associated with a decreased fertility of the previous AI, but it tended to increase calving/AI for multiparous cows with CL at NPD by decreasing the total pregnancy loss for the AI after treatment.

## Highlights

• G25&27 did not decrease nonpregnant cows without CL at day 32.
• G25&27 did not improve the overall fertility of primiparous cows.
• G25&27 was associated with a reduced P/AI of the previous AI in multiparous cows.
• G25&27 decreased pregnancy loss of AI after treatment in multiparous cows.
• G25&27 tended to increase calving per AI for AI after treatment in multiparous cows.

## Graphical Abstract

**Figure:**
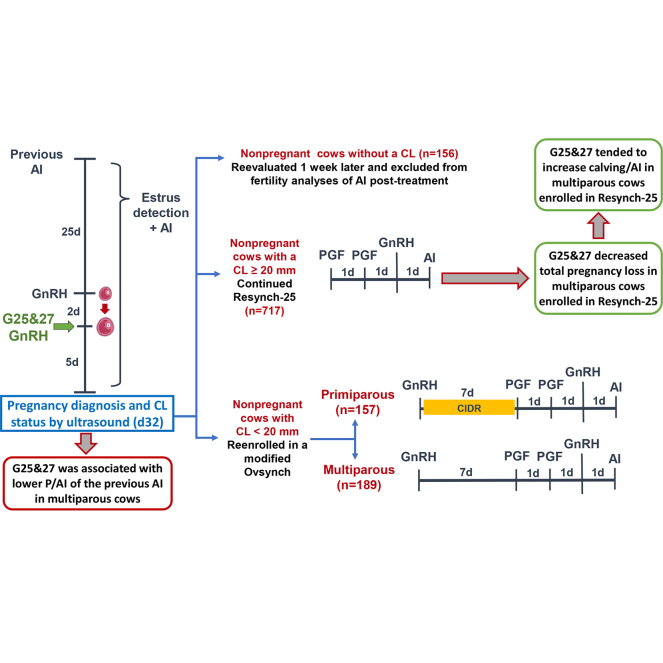
**Summary:** We investigated the effect of an additional gonadotropin-releasing hormone (GnRH) 2 days after initiation of resynchronization on day 25 post-artificial insemination (AI) on the proportion of cows without a corpus luteum (CL) at nonpregnancy diagnosis (NPD) and fertility of nonpregnant cows with CL at NPD that were reinseminated. Cows inseminated 25 days earlier were randomly assigned to either receive a GnRH only (control) or on days 25 and 27 (G25&27). Treatment did not improve overall fertility in primiparous cows. In multiparous cows, treatment was associated with a lower pregnancy per AI (P/AI) for the previous AI but tended to increase overall calving per AI after treatment because of the decrease in total pregnancy loss. Hence, the addition of a GnRH 2 days after the day-25 post-AI GnRH does not seem to be beneficial to improving the overall fertility of lactating dairy cows.

Minimizing the interval between inseminations (**IBI**) improves insemination risk and 21-d pregnancy rate of dairy herds (Overton and Cabrera, 2017). A simple approach to reduce IBI is to reinseminate nonpregnant cows detected in estrus after previous AI and before pregnancy diagnosis (Galvão et al., 2007). However, about 50% of nonpregnant cows do not show signs of estrus and reach pregnancy diagnosis (Giordano et al., 2015). For these cows, a timed-AI program can be used to reduce IBI. A common strategy implemented in US dairy farms for resynchronization of ovulation is to initiate Ovsynch 25 d after previous AI (**Resynch-25**; Silva et al., 2009).

A critical aspect of Ovsynch for achieving a greater P/AI is the ovulatory response to the first GnRH (**G1**). Ovulation to G1 controls the onset of the ovulatory follicular wave and improves responsiveness to all following treatments, resulting in better synchronization at timed-AI and pregnancy per AI (**P/AI**; Bello et al., 2006). Nevertheless, inducing ovulation of nonpregnant cows to G1 in a Resynch-25 program is challenging and has been reported to vary between 40% and 62% (Pérez et al., 2020; Leão et al., 2024).

The 2 main factors associated with ovulation failure to GnRH administration are high circulating progesterone (**P4**) concentrations (Colazo et al., 2008) and the absence of a dominant follicle responsive to an LH surge (Sartori et al., 2001). High circulating P4 concentrations reduce the magnitude of the LH surge from the anterior pituitary after GnRH administration (Giordano et al., 2012a). A larger GnRH dose (200 µg) effectively increased LH surge in cows with high circulating P4 concentration compared with a smaller GnRH dose (100 µg; Giordano et al., 2012a). In a recent study from our laboratory, increasing the dose of GnRH from 100 to 200 µg did not improve ovulatory response to G1 on d 25 post-AI in nonpregnant cows (Leão et al., 2024). This result provides evidence that an absence of a dominant follicle on d 25 post-AI may be the primary reason for a poor ovulatory response to GnRH. Considering a follicle's acquisition of ovulatory capability is an age-dependent characteristic (Lévy et al., 2000), administration of an additional GnRH 2 d after G1 may improve ovulatory response at the initiation of the resynchronization, resulting in an improved P/AI.

This study aimed to determine the effect of an additional GnRH treatment 2 d after the initiation of resynchronization on d 25 after previous AI on the proportion of cows without a corpus luteum (**CL**) at the time of nonpregnancy diagnosis (**NPD**), as well as on P/AI of cows with a CL at NPD that were reinseminated. Our main hypothesis is that nonpregnant cows receiving the extra GnRH on d 27 would have greater P/AI. Additionally, we hypothesized that fewer cows receiving the additional GnRH would be diagnosed without CL at NPD.

This experiment was conducted from August 2020 to January 2021 on 2 commercial farms in California. The average milk yield of the closest DHI test to treatment was 42.2 ± 0.2 kg/d for multiparous cows and 38.3 ± 0.2 kg/d for primiparous cows. Cows ranged from first to twelfth lactation and from second to eighth service at enrollment. In farm 1, all first-service cows were inseminated after Double Ovsynch at 75 ± 3 DIM (GnRH – 7 d – PGF_2α_ – 3 d – GnRH – 7 d – GnRH – 7 d – PGF_2α_ – 1 d – PGF_2α_ – 1 d – GnRH – 1 d – AI). Farm 2 used Presynch-12/Ovsynch initiated at 47 ± 3 DIM combined with AI following detection of estrus after the voluntary waiting period of 50 DIM for first service (PGF_2α_ – 14 d – PGF_2α_ – 12 d – GnRH – 7 d – PGF_2α_ – 1 d – PGF_2α_ – 1 d – GnRH – 1 d – AI). Artificial insemination was performed daily following estrous detection using tail paint on both farms for ≥second services.

Once per week, cows that received AI 25 d before (primiparous, n = 1,071; multiparous, n = 1,722) were randomly assigned to receive a GnRH treatment only (**G25**; primiparous, n = 525; multiparous, n = 877) or GnRH on this day and 2 d later (**G25&27**; primiparous, n = 546; multiparous, n = 845). Seven days after G1 (d 32), farm veterinarians performed pregnancy diagnosis and CL presence determination by transrectal ultrasonography (Easi-Scan, BCF Technology Ltd.). Cows that were detected in estrus and received AI (G25, n = 36; G25&27, n = 43) or were sold or died (G25, n = 15; G25&27, n = 15) between 25 and 32 d after previous AI were removed from the analysis. Cows with a CL and diagnosed as nonpregnant were enrolled in different resynchronization strategies based on parity (primiparous or multiparous) and CL diameter. This reproductive management strategy had already been implemented on the farms and could not be modified for the study. Multiparous cows with CL diameter <20 mm (n = 189) reinitiated a modified Ovsynch at NPD (d 32: GnRH – d 39: PGF_2α_ – d 40: PGF_2α_ – d 41: GnRH – d 42: timed-AI). Primiparous cows with CL diameter <20 mm (n = 157) also started a modified Ovsynch at NPD with an intravaginal progesterone device (CIDR, 1.38 g of P4, Eazi-Breed CIDR, Zoetis) inserted at NPD and removed 7 d later (Ovsynch+CIDR). Cows without a visible CL by ultrasound at NPD (n = 156) were reexamined 1 wk later and then enrolled in a resynchronization strategy according to the ovarian structures, as previously described. These cows were not included in the fertility analyses (i.e., P/AI, pregnancy loss, and calving/AI). At NPD, some cows were recorded as “do not breed” (n = 161) or with an abnormality by the veterinarian (n = 175) and were excluded from the analyses. The remaining cows had at least one CL with diameter ≥20 mm (primiparous, n = 287; multiparous, n = 457) and continued Resynch-25 at NPD (d 32: PGF_2α_ – d 33: PGF_2α_ – d 34: GnRH – d 35: AI). The post-treatment AI were performed by on-farm AI technicians (n = 8) using conventional Holstein (primiparous, n = 47; multiparous, n = 24) and Angus (primiparous, n = 186; multiparous, n = 433) semen from multiple sires. Primiparous cows inseminated with sexed semen (n = 27) were excluded from post-treatment fertility analyses. All multiparous cows were inseminated with conventional semen after treatment. Cows were allowed to be enrolled more than once, and if so, they were randomly assigned to one of the treatments again. Therefore, the experimental unit was each AI. The hormonal treatments used 86 µg of GnRH i.m. (Cystorelin; Boehringer Ingelheim, Duluth, GA) and 0.5 mg of cloprostenol i.m. for PGF_2α_ (SynchSure; Boehringer Ingelheim).

All statistical analyses were conducted using SAS (version 9.4; SAS Institute Inc., Cary, NC). All response variables were binary and analyzed using the GLIMMIX procedure. Variables are presented as proportions that were obtained using the MEANS procedure.

In the analysis of fertility traits of the AI before treatment, multiparous and primiparous cows were evaluated separately, because of the differences in the type of semen used for the different parity groups. Only 4 AI before treatment of multiparous cows used sexed semen, which were excluded from the analysis. Among the 1,061 AI before treatment of primiparous cows, 289 used sexed semen. Therefore, the models for multiparous cows considered the fixed effects of treatment (G25 or G25&27), service number (2, 3, 4, and >4), and AI technician, whereas, for primiparous cows, we added the fixed effect of semen type (sexed or conventional).

The model evaluating the proportion of cows without CL and with CL <20 mm as response variables considered treatment, parity (primiparous or multiparous), and service number as fixed effects. The analysis of fertility traits of cows that continued Resynch-25 used a similar model with the addition of the fixed effect of AI technician. For the analysis of fertility response variables in cows submitted to Ovsynch and Ovsynch+CIDR at NPD, the same model was used, excluding the fixed effect of parity. This exclusion was based on the separate analysis of primiparous and multiparous cows, as parity was a criterion for assignment to the resynchronization program. All models included farm as a random effect.

All possible 2-way interactions were tested in all models. Interactions with *P* > 0.20 were backward eliminated from the initial models. When possible, the interaction treatment × parity was forced in the final models to assess treatment effect within each parity group. The LSM statement was used to evaluate pairwise comparisons. Significance was considered when *P* ≤ 0.05 and tendency to significance when *P* > 0.05 and *P* ≤ 0.10.

Treatment was not associated with P/AI of the previous AI in primiparous cows (Table 1). Moreover, primiparous cows receiving sexed semen in the AI before treatment had a decreased (*P* < 0.01) P/AI compared with primiparous cows inseminated with conventional semen (sexed: 32.2%; conventional: 50.8%). This observation aligns with the anticipated pattern of P/AI using sexed semen that typically ranges from 70% to 90% of that achieved with conventional semen in lactating dairy cows (Karakaya-Bilen et al., 2019).

**Table 1:** Effect of treatment (Treat) on pregnancy per AI (P/AI) and pregnancy loss of the AI before treatment

Relative to AI before treatment	G25^1^	G25&27^2^	*P*-value		
			Treat	Service number	Semen type
Primiparous cow					
P/AI on d 32, % (n/n)	46.6 (243/521)	44.8 (242/540)	0.56	<0.01	<0.01
P/AI on d 102, % (n/n)	40.5 (207/511)	39.3 (207/527)	0.82	0.02	0.02
Pregnancy loss between d 32 and 102, % (n/n)	11.2 (26/233)	9.6 (22/229)	0.45	0.17	0.17
Multiparous cow					
P/AI on d 32, % (n/n)	42.4 (366/863)	36.6 (306/835)	0.01	0.78	—
P/AI on d 102, % (n/n)	34.2 (287/839)	29.3 (237/808)	0.03	0.83	—
Pregnancy loss between d 32 and 102, % (n/n)	16.1 (55/342)	15.1 (42/279)	0.77	0.90	—

1 Cows treated with GnRH on d 25 after previous AI.

2 Cows treated with GnRH on d 25 and 27 after previous AI.

Surprisingly, G25&27 was associated with a smaller P/AI to the AI before treatment in multiparous cows (Table 1). In a study with a limited number of cows, Moreira et al. (2000) reported a likely negative association (*P* = 0.08) of a single GnRH treatment on d 20 for pregnancy loss between d 20 and 27 post-AI. Nevertheless, other subsequent studies showed no adverse effect of a single GnRH treatment, 2 GnRH treatments 7 d apart, or human chorionic gonadotropin (hCG) combined with GnRH 7 d apart (d 18 and d 25 post-AI) in cows with unknown pregnancy status on P/AI of previous AI (Chebel et al., 2003; Fricke et al., 2003; Giordano et al., 2012b). We are not aware of any previous study using a resynchronization strategy with 2 consecutive GnRH treatments 2 d apart for lactating dairy cows with unknown pregnancy status. The additional GnRH treatment (d 27 post-AI) in our study design was administered in cows at the same time of the day as the last GnRH of Ovsynch when cows of the same pen were already locked in self-locking stanchions. Therefore, no extra locking time was expected for cows in G25&27. Still, cows in G25&27 received one more i.m. injection than G25 2 d later, which may be an extra physical stress factor for cows. Interestingly, this negative association between the extra GnRH and P/AI of the AI preceding treatment was not observed in primiparous cows.

Furthermore, we did not expect that G25&27 treatment would increase ovulatory response in pregnant cows. A recent study from our laboratory indicated that the ovulatory response of pregnant cows receiving a GnRH 25 d after AI is approximately 13% (Leão et al., 2024). This small proportion of animals that ovulate to this GnRH treatment would probably not significantly affect P/AI of the previous AI. Finally, we did not observe an effect of treatment on pregnancy losses between d 32 and d 102 after the AI before treatment in primiparous or multiparous cows (Table 1).

Different from what we hypothesized, treatment was ineffective (*P* = 0.58) in decreasing the overall proportion of nonpregnant cows without a CL at d 32 NPD (G25: 13.5%; G25&27: 12.7%). Our previous study that compared 2 doses of GnRH (100 vs. 200 µg) on d 25 after previous AI reported a similar proportion of nonpregnant cows without a CL at d 32 NPD (13.1%, n = 1,437; Leão et al., 2024). Although the proportion of cows without CL did not differ between treatments in the present study, treatment tended (*P* = 0.09) to increase the overall proportion of cows with CL <20 mm in diameter at NPD (G25: 26.7%; G25&27: 31.4%). Cows without a CL that ovulated to the d 27 GnRH would have a 5-d-old CL at the NPD, which most likely had a diameter <20 mm (Cunha et al., 2022). Although we did not evaluate ovulatory response to GnRH on d 25 and d 27, the present results of CL status at NPD suggest that the extra GnRH on d 27 did not improve overall ovulatory response at the start of resynchronization.

For multiparous cows that had a CL >20 mm and continued Resynch-25 at NPD, the additional GnRH tended (*P* = 0.06) to decrease pregnancy loss between 32 and 102 d. This group of cows also had decreased (*P* < 0.01) total pregnancy loss (from d 32 to calving), leading to a tendency (*P* = 0.08) to increase calving rate for the G25&27 group compared with G25 in multiparous cows (Table 2). We speculated that 2 factors may have contributed to these results. The first factor may be an increased ovulatory response at the initiation of Ovsynch that is associated with better fertility (Pérez et al., 2020). The second is a potentially smaller proportion of twin pregnancies, which probably explains the fewer pregnancy losses. Even though G25&27 did not decrease the proportion of cows with no CL at NPD, we speculate that a greater proportion of cows in this group might have formed an accessory CL at the beginning of the protocol. Research has shown that the formation of an accessory CL increases serum P4 concentration (Cunha et al., 2022), decreasing the chances of selection and ovulation of multiple follicles and, consequently, twin pregnancies (Wiltbank et al., 2014). High-producing multiparous cows are more susceptible to twin pregnancies (Schambow et al., 2021) because of an increased P4 catabolism (Wiltbank et al., 2014). Reducing twin pregnancies would likely decrease pregnancy losses. This hypothesis is reinforced because G25&27 only decreased the total pregnancy loss in multiparous cows, which are more likely to become pregnant with twins (Schambow et al., 2021). Yet, the limited number of pregnancies and calvings for cows with CL >20 mm in this study lead to insufficient statistical power and a high probability of type II error for the pregnancy loss and twinning results.

**Table 2:** Effect of treatment (Treat) on pregnancy per AI (P/AI), calving per AI, and pregnancy loss in primiparous and multiparous cows with corpus luteum (CL) >20 mm in diameter at nonpregnant diagnosis submitted to the Resynch-25 protocol for resynchronization of ovulation

Item	Primiparous		Multiparous		*P*-value			
	G25^1^	G25&27^2^	G25^1^	G25&27^2^	Treat	Parity	Service number	Treat × parity
P/AI on d 32, % (n/n)	44.7^A^ (51/114)	42.9^AB^ (48/112)	36.1^B^ (78/216)	35.5^B^ (78/220)	0.28	0.04	0.26	0.70
P/AI on d 102, % (n/n)	37.8^a^ (42/111)	35.5^ab^ (38/107)	26.2^b^ (54/206)	29.4^b^ (63/214)	0.32	0.02	0.20	0.31
Calving rate, % (n/n)	34.3^a^ (36/105)	35.2^a^ (37/105)	19.8a,b, A,B (38/192)	27.0a,b, A,B (55/204)	0.30	<0.01	0.28	0.22
Pregnancy loss between d 32 and 102, % (n/n)	12.5^B^ (6/48)	11.6^B^ (5/43)	23.5^A^ (16/68)	12.5^B^ (9/72)	0.26	0.26	0.91	0.28
Pregnancy loss between d 102 and calving, % (n/n)	2.7 (1/37)	2.6 (1/38)	11.6 (5/43)	1.8 (1/56)	0.20	0.89	0.50	0.43
Pregnancy loss between d 32 and calving, % (n/n)	16.3^a^ (7/43)	14.0^a^ (6/43)	36.8^b^ (21/57)	15.4^a^ (10/65)	0.06	0.13	0.95	0.14
Twin birth, % (n/n)	5.6 (2/36)	8.1 (3/37)	7.9 (3/38)	3.6 (2/55)	0.81	0.64	0.55	0.34

a,b Means with different superscripts within a row differ (*P* ≤ 0.05).

A,B Means with different superscripts within a row tend to differ (0.10 > *P* > 0.05).

1 Cows treated with GnRH on d 25 after previous AI.

2 Cows treated with GnRH on d 25 and 27 after previous AI.

Primiparous cows with a CL <20 mm and submitted to Ovsynch+CIDR at NPD in the G25&27 treatment tended to have decreased P/AI 102 d after AI (*P* = 0.07) and reduced calving/AI (*P* = 0.08; Table 3). This tendency is probably related to the stage of follicular development at G1 of the Ovsynch+CIDR protocol (d 32). Cows that ovulated to the second GnRH treatment would have an approximately 4-d-old follicle at G1, which most likely has not developed ovulatory capability because of the absence of LH receptors in the granulosa cells (Xu et al., 1995). In these cows, the lack of ovulatory response to G1 with the addition of a CIDR might have extended the follicular dominance period, promoting a persistent follicle that leads to lower-quality oocytes (Cerri et al., 2009; Geary et al., 2013). These oocytes are more likely to undergo premature nuclear maturation (Revah and Butler, 1996), resulting in less viable embryos (Cerri et al., 2009).

**Table 3:** Effect of treatment (Treat) on pregnancy per AI (P/AI), calving per AI, and pregnancy loss of cows with corpus luteum (CL) with diameter <20 mm at nonpregnancy diagnosis submitted to different strategies for resynchronization on d 32 after previous AI

Relative to AI after treatment	G25^1^	G25&27^2^	*P*-value	
			Treat	Service number
Primiparous cows enrolled in Ovsynch + CIDR on d 32				
P/AI on d 32, % (n/n)	49.2 (32/65)	40.5 (30/74)	0.34	0.48
P/AI on d 102, % (n/n)	45.5 (30/66)	29.5 (23/78)	0.07	0.53
Calving per AI, % (n/n)	42.6 (26/61)	28.8 (21/73)	0.08	0.54
Pregnancy loss between d 32 and 102, % (n/n)	6.5 (2/31)	18.5 (5/27)	0.14	0.78
Twin birth, % (n/n)	3.8 (1/26)	9.5 (2/21)	0.58	0.99
Multiparous cows enrolled in Ovsynch on d 32				
P/AI on d 32, % (n/n)	31.6 (24/76)	27.8 (25/90)	0.36	0.11
P/AI on d 102, % (n/n)	26.7 (20/75)	24.7 (22/89)	0.72	0.26
Calving per AI, % (n/n)	24.7 (18/73)	22.1 (19/86)	0.63	0.46
Pregnancy loss between d 32 and 102, % (n/n)	13.0 (3/23)	8.3 (2/24)	0.99	0.81
Twin birth, % (n/n)	22.2 (4/18)	10.5 (2/19)	0.09	0.74

1 Cows treated with GnRH on d 25 after previous AI.

2 Cows treated with GnRH on d 25 and 27 after previous AI.

In contrast to the observations made for primiparous cows enrolled in the Ovsynch+CIDR protocol on d 32, the treatment had no effect on the fertility of multiparous cows with CL <20 mm enrolled in Ovsynch at NPD. The absence of a CIDR in the resynchronization program of multiparous cows confounds the interpretation of the different treatment results observed in primiparous cows compared with multiparous cows with CL <20 mm. Other physiological factors may also have contributed to this. For instance, multiparous cows are more likely to lose pregnancies in the embryonic stage of development than primiparous cows (Quintero Rodríguez et al., 2019), which can prolong the IBI (Remnant et al., 2015). Still, without data on follicle status and other physiological parameters during and after treatment, these results are difficult to interpret. In addition, the limited number of primiparous and multiparous cows with CL <20 mm reduces the statistical power and increases the chance of type II errors for the fertility parameters analyses.

We also analyzed P/AI from all cows regardless of the strategy adopted at NPD to evaluate the overall treatment effect for the AI post-treatment. In these analyses, we did not observe a treatment effect on the fertility of primiparous cows. Yet, multiparous cows in the G25&27 group tended (*P* = 0.08) to have increased calving rate (G25: 25.3%; G25&27: 31.3%) and decreased (*P* = 0.06) pregnancy loss between d 32 and 102 (G25: 18.0%; G25&27: 10.3%), and decreased (*P* < 0.01) total pregnancy loss (G25: 27.1%; G25&27: 13.0%).

In summary, our results showed that G25&27 was associated with decreased fertility to the AI before treatment in multiparous cows. For AI after treatment, G25&27 tended to increase calving/AI in multiparous cows because of a decreased total pregnancy loss. This same treatment did not affect overall fertility in primiparous cows but tended to reduce P/AI on d 102 post-AI and calving rate of primiparous cows with a CL <20 mm and enrolled in Ovsynch+CIDR at NPD.

## References

[bib1] Bello N.M., Steibel J.P., Pursley J.R. (2006). Optimizing ovulation to first GnRH improved outcomes to each hormonal injection of Ovsynch in lactating dairy cows. J. Dairy Sci..

[bib2] Cerri R.L.A., Rutigliano H.M., Chebel R.C., Santos J.E.P. (2009). Period of dominance of the ovulatory follicle influences embryo quality in lactating dairy cows. Reproduction.

[bib3] Chebel R.C., Santos J.E.P., Cerri R.L.A., Galvão K.N., Juchem S.O., Thatcher W.W. (2003). Effect of resynchronization with GnRH on day 21 after artificial insemination on pregnancy rate and pregnancy loss in lactating dairy cows. Theriogenology.

[bib4] Colazo M.G., Kastelic J.P., Davis H., Rutledge M.D., Martinez M.F., Small J.A., Mapletoft R.J. (2008). Effects of plasma progesterone concentrations on LH release and ovulation in beef cattle given GnRH. Domest. Anim. Endocrinol..

[bib5] Cunha T.O., Statz L.R., Domingues R.R., Andrade J.P.N., Wiltbank M.C., Martins J.P.N. (2022). Accessory corpus luteum induced by human chorionic gonadotropin on day 7 or days 7 and 13 of the estrous cycle affected follicular and luteal dynamics and luteolysis in lactating Holstein cows. J. Dairy Sci..

[bib6] Fricke P.M., Caraviello D.Z., Weigel K.A., Welle M.L. (2003). Fertility of dairy cows after resynchronization of ovulation at three intervals following first timed insemination. J. Dairy Sci..

[bib7] Galvão K.N., Santos J.E.P., Cerri R.L., Chebel R.C., Rutigliano H.M., Bruno R.G., Bicalho R.C. (2007). Evaluation of methods of resynchronization for insemination in cows of unknown pregnancy status. J. Dairy Sci..

[bib8] Geary T.W., Smith M.F., MacNeil M.D., Day M.L., Bridges G.A., Perry G.A., Abreu F.M., Atkins J.A., Pohler K.G., Jinks E.M., Madsen C.A. (2013). Triennial Reproduction Symposium: Influence of follicular characteristics at ovulation on early embryonic survival. J. Anim. Sci..

[bib9] Giordano J.O., Fricke P.M., Guenther J.N., Lopes G., Herlihy M.M., Nascimento A.B., Wiltbank M.C. (2012). Effect of progesterone on magnitude of the luteinizing hormone surge induced by two different doses of gonadotropin-releasing hormone in lactating dairy cows. J. Dairy Sci..

[bib10] Giordano J.O., Stangaferro M.L., Wijma R., Chandler W.C., Watters R.D. (2015). Reproductive performance of dairy cows managed with a program aimed at increasing insemination of cows in estrus based on increased physical activity and fertility of timed artificial inseminations. J. Dairy Sci..

[bib11] Giordano J.O., Wiltbank M.C., Guenther J.N., Ares M.S., Lopes G., Herlihy M.M., Fricke P.M. (2012). Effect of presynchronization with human chorionic gonadotropin or gonadotropin-releasing hormone 7 days before resynchronization of ovulation on fertility in lactating dairy cows. J. Dairy Sci..

[bib12] Karakaya-Bilen E., Yilmazbas-Mecitoglu G., Keskin A., Guner B., Serim E., Santos J.E.P., Gumen A. (2019). Fertility of lactating dairy cows inseminated with sex-sorted or conventional semen after Ovsynch, Presynch-Ovsynch and Double-Ovsynch protocols. Reprod. Domest. Anim..

[bib13] Leão I.M.R., El Azzi M.S., Anta-Galvan E., Valdes-Arciniega T., Martins J.P.N. (2024). Effects of 200 µg of gonadorelin at the first gonadotropin-releasing hormone of the Resynch-25 on ovarian dynamics and fertility in lactating Holstein cows. J. Dairy Sci..

[bib14] Lévy D.P., Navarro J.M., Schattman G.L., Davis O.K., Rosenwaks Z. (2000). The role of LH in ovarian stimulation: Exogenous LH: Let's design the future. Hum. Reprod..

[bib15] Moreira F., Risco C.A., Pires M.F.A., Ambrose J.D., Drost M., Thatcher W.W. (2000). Use of bovine somatotropin in lactating dairy cows receiving timed artificial insemination. J. Dairy Sci..

[bib16] Overton M.W., Cabrera V.E., Beede D. (2017). Large Dairy Herd Management.

[bib17] Pérez M.M., Wijma R., Scarbolo M., Cabrera E., Sosa F., Sitko E.M., Giordano J.O. (2020). Lactating dairy cows managed for second and greater artificial insemination services with the Short-Resynch or Day 25 Resynch program had similar reproductive performance. J. Dairy Sci..

[bib18] Quintero Rodríguez L.E., Rearte R., Domínguez G., Luzbel de la Sota R., Madoz L.V., Giuliodori M.J. (2019). Late embryonic losses in supplemented grazing lactating dairy cows: Risk factors and reproductive performance. J. Dairy Sci..

[bib19] Remnant J.G., Green M.J., Huxley J.N., Hudson C.D. (2015). Variation in the interservice intervals of dairy cows in the United Kingdom. J. Dairy Sci..

[bib20] Revah I., Butler W.R. (1996). Prolonged dominance of follicles and reduced viability of bovine oocytes. Reproduction.

[bib21] Sartori R., Fricke P.M., Ferreira J.C.P., Ginther O.J., Wiltbank M.C. (2001). Follicular deviation and acquisition of ovulatory capacity in bovine follicles. Biol. Reprod..

[bib22] Schambow R.A., Bennett T.B., Döpfer D., Martins J.P.N. (2021). A retrospective study investigating the association of parity, breed, calving month and year, and previous parity milk yield and calving interval with twin births in US dairy cows. J. Dairy Sci..

[bib23] Silva E., Sterry R.A., Kolb D., Mathialagan N., Mcgrath M.F., Ballam J.M., Fricke P.M. (2009). Effect of interval to resynchronization of ovulation on fertility of lactating Holstein cows when using transrectal ultrasonography or a pregnancy-associated glycoprotein enzyme-linked immunosorbent assay to diagnose pregnancy status. J. Dairy Sci..

[bib24] Wiltbank M.C., Souza A.H., Carvalho P.D., Cunha A.P., Giordano J.O., Fricke P.M., Baez G.M., Diskin M.G. (2014). Physiological and practical effects of progesterone on reproduction in dairy cattle. Animal.

[bib25] Xu Z., Garverick H.A., Smith G.W., Smith M.F., Hamilton S.A., Youngquist R.S. (1995). Expression of follicle-stimulating hormone and luteinizing hormone receptor messenger ribonucleic acids in bovine follicles during the first follicular wave. Biol. Reprod..

